# A scoping review on the use of reflection and reflective portfolio learning in veterinary education

**DOI:** 10.1002/vro2.79

**Published:** 2024-05-07

**Authors:** Andrea Jones, Kate Cobb, Gary England

**Affiliations:** ^1^ University of Nottingham Sutton Bonington Campus Sutton Bonington UK

## Abstract

**Introduction:**

In veterinary education, reflection and reflective portfolio learning aim to enhance professional development. Although reflection and reflective portfolio learning are widely used in teaching and healthcare, their demonstrable impact on veterinary education is unclear. Although the benefits are uncertain, reflection may provide potential for self‐development and help to prepare students for clinical practice. The aim of this work was to review research into reflective practice and reflective portfolio learning, to find evidence for its use in teaching, to confirm how it is best implemented to optimise professional development and to identify gaps for future research.

**Methods:**

This scoping review was conducted according to the Joanna Briggs Institute guidelines adhering to review methodology. Ten databases were searched and screened for reflection and portfolio learning as a primary source of data. Of 29,933 texts identified by title and/or abstract, 51 final works were screened in full. Of these, 16 papers that gathered evidence on reflection were included.

**Results:**

The results confirmed a growing evidence base for reflection in the veterinary field. In total, six works demonstrated a positive impact on professional development. Written reflective assignments were the most common methods of implementation, with 11 papers referencing their use. Other methods included group discussion and facilitated reflection.

**Conclusions:**

This review identified numerous gaps for research, including validating the methods of implementation, exploring the efficacy of methods other than written reflection, examining how reflection is used at different stages of the veterinary career and study of different models of reflection to identify which are most useful.

## INTRODUCTION

Reflection is considered an essential skill for professional practice and, for UK veterinary graduates, it is a day one competency.^1^ Reflection can allow recognition of strengths and weaknesses, identification of gaps in knowledge and provide motivation to learn. It may assist navigation through difficult communications and challenging decision making which can improve conflict management.^2^, ^3^ Establishing reflective skills throughout the undergraduate degree can prepare students for reflection‐in‐action,^4^ the ‘thinking on their feet’ required for practice. Schön discussed reflection as the way a professional might solve problems through practice by uncovering implicit knowledge to make sense and meaning, often with others.^4^ Reflection can develop resilience by increasing acceptance of uncertainty,^5^ which benefits patients and clients.

In reflective portfolios, students record learning experiences in a reflective writing exercise, thereby fulfilling the experiential learning cycle.^6^ Usually, this is an online record for assessing workplace skills. Reflective portfolios can be restrictive or open in terms of what is included, while the main aim in veterinary curricula is development towards day one competencies.^1^

There is a large amount of theoretical support for reflective practice in higher education and healthcare^4^, ^7^, ^8^, ^9^, ^10^; although it is unclear the extent of empirical evidence to support the use of reflection and reflective portfolio learning, specifically within veterinary education, is unclear. Scoping reviews seek to understand the extent of knowledge in a certain field, whereas systematic reviews inform decision making or policy.^11^ The purpose of this scoping review was to find evidence for use of reflection and reflective portfolio learning in veterinary education programmes, to understand the potential impact these interventions have on professional development and to find gaps for future research. A scoping review is an appropriate tool because it explores and maps literature and identifies gaps in research. A review in the veterinary area is warranted because the differences between human doctors/physicians (medics) and veterinary surgeons (vets) are sufficiently important to justify investigation in our area alone. There may be potential differences in the way vets and medics reflect. There are important normative differences between vets and medics in the ethical areas of patient care, owner communications, end‐of‐life care, euthanasia and relationships of trust, for all these areas undergraduate reflective exercises aim to prepare students to navigate them once graduated. Although extrapolation from healthcare is useful, due to these differences, it is important to have specific veterinary evidence.

The following questions were formulated for this study.

1. What evidence of a positive benefit exists to support the use of reflection and reflective portfolio learning in veterinary education?
2. Is there substantiated guidance on how best to implement reflection and reflective portfolio learning in a veterinary curriculum to optimise professional development?
3. What gaps exist in the evidence, and what should be the focus of future research regarding reflection and reflective portfolio learning?

## METHODS

Initial scoping searches were conducted between May 2021 and September 2021 to ascertain available evidence and to formulate the final search strategy. Using the Joanna Briggs Institute guidance document,^11^ systematic searches were conducted from October 2021 to March 2022 using keywords (Box 1) and subject headings where available within the concept areas of veterinary reflection and portfolio learning. A systematic literature search of 10 online databases was performed to find publications from all dates. The following databases were searched: PubMed, Education Resources Information Centre, Medline, CABabstracts, Web of Science, PsychINFO, Embase, Proquest Natural Science, Proquest Education and Scopus. The titles, or titles and abstracts, from 29,933 papers were screened for relevance. Review papers were screened to check for further references. An example search of PubMed is shown in Box 1.

**BOX 1: Example search of PubMed**

(‘Education, Veterinary’[Mesh] AND refle* (OR portfolio OR clerkship OR internship OR reside* OR ‘workplace based assessment’ OR profession* OR apprentice* OR ‘transition to practice’ OR transition OR ‘graduate attributes’ OR readiness OR employability OR ‘transferrable skills’)

### Eligibility criteria

Inclusion and exclusion criteria were developed to include veterinary or veterinary education context only, undergraduate or postgraduate, all available years of publication, any country and peer‐reviewed primary research with qualitative, quantitative data or mixed methods. The aim was to search for primary research on reflective practice and reflective portfolio learning in the veterinary setting. This information is displayed in Table 1. Papers not in English, grey literature, non‐peer reviewed and opinion/theory pieces (theoretical articles or narratives written by veterinary education experts with no primary data collected) were excluded.

**TABLE 1:** Study inclusion and exclusion criteria (based on O'Flaherty et al.^46^).

Criteria	Inclusion	Exclusion
Time scale	All years	All included
Article type	Peer‐reviewed original research with specifically measured data which is in English	Grey literature, conference proceedings, letters, commentaries, opinion pieces in another language
Population	Undergraduate and post graduate veterinary students, veterinary educators and experienced veterinarians.	Any other discipline, for example, medics, teachers of other subjects
Literature focus	Work that has evaluation of reflection or reflective portfolio work as the main application	Work that mentions reflection, or it is used as the tool to develop other attributes but is not the main focus of the work

### Theoretical background

There may be theoretical contradictions with a review of this subject. Most studies on reflection have an interpretive qualitative component. Reflection is subjective to individuals and is based on their experience and researcher interpretation. In this review, the approach endeavoured to remain true for positivistic methodology_._^11^

## RESULTS

### Numerical summary

**FIGURE 1:**
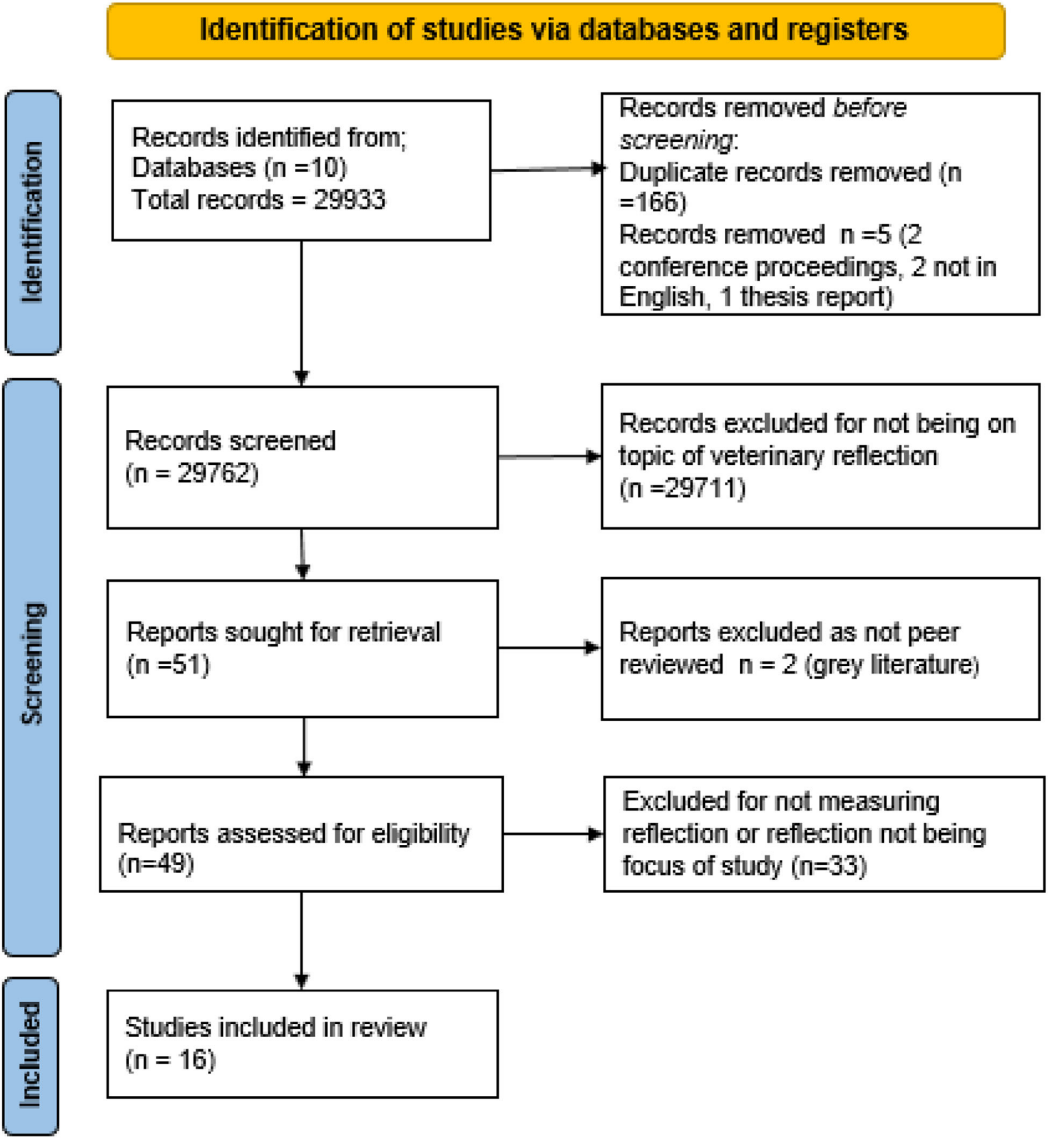
Methods flow chart—application of inclusion and exclusion criteria.^12^

Publications (*n* = 29,933) were screened by title and/or abstract, 51 relevant publications remained that were screened by full text, and 16 were included in the scoping review (Figure 1). Reasons for exclusion included 26 publications because evaluation of reflection was not the focus, four were opinion or theoretical pieces, three were not primary research (review papers) and two were grey literature. Of the final 16 publications evaluating reflection in veterinary medicine, nine were qualitative and seven were mixed methods. Eleven studies evaluated students’ reflective written assignments and five studies evaluated reflective discussion. The results are tabulated in Table S1.

### Narrative summary

#### Evidence of a positive effect on professional development

Altogether, six publications constituted a modest amount of evidence of a positive benefit for professional development via the reflective process,^3^, ^13^, ^14^, ^15^, ^16^, ^17^ with apparent value to reflection for professional practice. It was reported that reflective assignments could be used to assess and develop communication skills among veterinary undergraduates.^3^ These participants were instructed to reflect on communication, which may account for them not perceiving a use for reflection in other areas of professionalism.^3^ Vet and medic assignments were not directly comparable since although veterinarians were prompted to consider self‐awareness, medics were not.^3^ As the methods for participant groups were not directly comparable, it may be simpler to consider as two separate studies on the different participant groups. The difference in assignments emphasises the need to give considered instructions to students so that they have a chance to achieve intended outcomes. This may mean that there are differences between vets and medics regarding reflection, highlighting the need to research for evidence specific to the veterinary field. One study reported that reflection was useful for observing the views of others when considering animal welfare in undergraduates.^16^

A mixed methods study reported that reflective discussion groups helped to foster openness about mistakes in qualified vets, which the authors associated with the development of increased trust.^17^ Although qualitatively the participants in the reflective discussion groups perceived their reflective levels to increase, the authors did not observe this.^17^ Despite this lack of quantitative improvement in levels of reflection, increased openness can be considered a positive benefit of these discussions due to gains in practice culture.

Three publications reported that for postgraduates, reflection had a positive effect on professionalism.^13^, ^14^, ^15^ These three publications used postgraduate reflective essays from the UK Certificate in Advanced Veterinary Practice as evidence.^18^ The first publication reported that reflection produced a change in behaviour that positively affected individuals in their job and wider stakeholders.^13^ Other broad benefits for the reflective process were claimed, including communication, time management, self‐efficacy, reasoning, teamwork, motivation, job satisfaction, reduced stress, improved animal welfare and veterinarian effectiveness.^13^ These benefits may be interpreted as having a positive impact on the reflective process. The second publication reported that reflection fostered better stress‐coping mechanisms and understanding of the job.^14^ A better understanding of one's job and the ability to cope with stress could help with longevity in a veterinary career; similarly, this could be interpreted as a positive benefit for reflection. The third publication reported that reflection helped identify challenges in their work as clinicians.^15^ This could be considered another positive benefit for reflection because increased awareness may help recognise job fit for oneself and help progression towards job satisfaction. Finding an understanding of the contextual reality of general practice might release pressure on individuals. Understanding that things do go wrong and seldom are conditions exactly as we wish might ease decision making. Perhaps moving from striving for perfection to finding the best fit for patients and clients in that situation within the confines of available resources.

#### How were reflection and reflective portfolio learning implemented within a veterinary curriculum?

Reflection has been implemented in veterinary curricula in a variety of ways, including written assignments following video analysis,^3^ peer group discussion,^17^, ^19^, ^20^ facilitated discussion groups,^21^ reflective essays,^13^, ^14^, ^15^, ^22^, ^23^ frame reflection (reflecting by considering another's viewpoint),^16^ a method utilising Kolb's Experiential Learning Theory (ELT) (a cycle of learning by experience and reflection),^21^ reflective portfolio creation^24^ and an approach using artistic methods such as creative writing and modelling.^25^ One publication reported that reflective group work was useful for facilitating attainment of critical reflection.^19^

Eleven veterinary studies reported using written work^3^, ^13^, ^14^, ^15^, ^16^, ^19^, ^21^, ^23^, ^24^, ^25^, ^26^ to implement reflection, with one confirming successful development of reflective ability via essays in postgraduate teachers.^22^ Reflective writing may be used most widely to provide the necessary structure for reflective assignments. Frame reflection has also been used as a tool to increase appreciation of values and opinions of others, which could assist with ethical decision making where there is a need to consider all stakeholders.^16^ Creative methods such as writing verse, composing music, drawing and modelling can help develop reflection in some individuals.^25^ Although attaining critical levels of reflection is not a guaranteed outcome of creative methods, it could be a useful adjunct for some students.^25^ The use of more formal models, such as Kolb's^6^ experiential learning cycle, has also demonstrated positive impacts on reflective ability.^21^

Although there was initial reluctance, at the postgraduate level, reflective essays have been found to positively impact workplace behaviours,^13^ with preceptor input helping to develop reflective ability.^22^ Whichever way reflective ability is learned and assessed, there is concern over judgement and honest reflection.^24^ Assessment of clinical competence could be seen as contradictory to the outcomes of honest critical self‐reflection, although acknowledging weakness is also an important part of competency.

In contrast to the wealth of evidence in teaching and medical education, there was only one study^24^ on reflective portfolio learning in veterinary education. That study found that the portfolio format was a barrier to reflection.^24^ It reported on students’ experience with reflective portfolio learning and assessment using focus groups with the aim of identifying barriers to reflection.^24^ It was acknowledged that students may have difficulty accepting portfolio learning.^24^ Portfolio data could be less useful because the attributes developed are important and the portfolio format varies greatly between contexts, with different levels of reflection utilised. However, the use of reflective portfolios is extensive within veterinary education; therefore, further research is warranted to better understand how they can be used to enhance reflective ability and professional development.

Models for reflection have been used to varying degrees of success, with Kolb's ELT^6,21^ and concept mapping found to be useful.^26^ Conversely, some studies found models lacking^3^, ^24^ and perhaps less useful for developing critical reflections.^25^ Some studies reported that students had initial reticence for reflective learning,^15^, ^24^ resistance to reflective writing or reliving negative events and an understandable fear of being judged.^27^

### Gaps in the published literature

Sixteen publications contributed to the growing evidence base for reflection in the veterinary area, nine were qualitative^13^, ^14^, ^15^, ^22^, ^23^, ^24^, ^25^, ^27^, ^28^ and seven used mixed methods.^3^, ^16^, ^17^, ^19^, ^20^, ^21^, ^26^ This qualitative evidence identifies experiences and insights that could be transferable across different contexts, with the caveat that the evidence is exploratory and specific to the context in which it was carried out. However, findings from these studies may be transferrable and used to inform practice within veterinary education and the profession more broadly.

There are several gaps in the literature. Building on current work, there are opportunities to continue to develop more evidence that reflection has a useful effect and that it promotes professionalism in different contexts and educational settings. With much of the evidence utilising essays as a reflective tool, this and other methods of reflective practice could be explored and validated. With little evidence for methods of implementation, there is opportunity to confirm the value of written reflection and investigate whether different types of reflection would be useful, such as group or spoken. Currently, for group reflection, there is some useful evidence for veterinary educators, with this format helping achieve critical reflection.^19^ In practising vets, reflective discussion groups led to positive changes in workplace culture, fostering a more open environment where error is acknowledged and discussed, leading to improved patient outcomes.^17^ However, group discussion must be encouraged in a safe environment, where the focus is not to apportion blame, for this to be successful. Furthermore, new graduates may need this form of reflection to be formalised rather than ad hoc discussions in the workplace.^28^

Other gaps to elucidate include how reflection is best implemented for all veterinary career stages and how to promote undergraduate engagement with reflective portfolio learning. A similar gap exists to work with students studying reflection at different levels, such as those beginning or developing their reflective ability. Finally, further work on support and guidance for educators to deliver and assess veterinary reflection would be a useful addition to the current evidence base.

## DISCUSSION

This scoping review revealed some empirical evidence demonstrating a positive benefit for reflection and reflective portfolio learning in veterinary education, with seven of 16 publications demonstrating a positive effect on professionalism.^3^, ^13^, ^14^, ^15^, ^16^, ^17^, ^19^ The use of reflection in veterinary education has been shown to positively impact communication skills,^3^, ^16^, ^21^ reduce workplace stress levels,^14^ increase awareness of an individual's role, improve teamwork^14^ and increase understanding of values and opinions of others.^16^ The evidence base is fairly small, contextually situated and often based on qualitative studies. However, as aforementioned, these may be transferrable across the profession. Although reflective essays are the most commonly used tool described in the studies included in this review,^3^, ^13^, ^14^, ^15^, ^22^, ^23^, ^25^ interestingly, other methods, including small group discussions, were shown to be beneficial.^19^, ^20^ Written work may be most common due to interpreted alignment with early reflection, which requires a purposive approach.^29^ Therefore, there is a need for further research to add to the current evidence on methods of implementation and to explore the benefits and challenges of different models and tools for developing reflective practice.

Being incorporated into veterinary education since 2006,^3^ and slightly later in the UK,^30^ it is surprising that there has been limited evaluation of veterinary reflection. Despite this lack of evidence, due to perceived benefits, reflection has permeated all areas and stages of veterinary careers, from students to educators. With evidence for resistance to this learning and assessment from students^24^ and how challenging it is to teach and grade,^22^ there is a requirement to continue empirical consideration of this concept in veterinary education. Existing exploratory work is useful because it helps in understanding and defining reflection for vets,^25^ among learners and educators. Students need to know explicitly what they are being assessed on and educators require standardisation to align teaching and assessment.^31^

It is challenging to assess^22^ and be assessed on reflection,^23^ due to its qualitative nature, it is understood and graded differently by individuals.^22^ There is some understandable resistance to being judged on authentic personal reflections.^24^ Students may also misunderstand what is required and thinking about an emotional account or stepping outside professional boundaries is necessary.^25^ This may have implications for welfare if students feel they have been more open than required for little gain. Being assessed on reflection has the potential to precipitate fake reflections to score highly or deliver what assessors might be looking for.^32^ Creating a tick box approach rather than truly reflecting for professional development. This leads to a question of whether it is useful to grade reflections or should it be sufficient to show committed and authentic reflective development viz. Schön's reflective practitioner.^4^ Despite these complications, there is scope to continue to elicit more detail on reflection, exploring its positive effect further and how it can be operationalised in educative or clinical practice.

Reflection is one of the guiding theoretical frameworks of professionalism education, linked to experiential learning and part of developing professional identity.^33^, ^34^ Becoming a professional requires the student to experience veterinary duties for themselves, putting learning into practice so they can begin to understand how all stakeholders (including themselves) react to contextual complexities.^35^ Analysing these encounters allows students to understand their position with regards to progression, promoting life‐long learning required to keep up with professional development throughout their career.^36^ Ideally, this academic thought develops broadmindedness, self‐awareness and reflective problem‐solving abilities.^29^

Currently, theory frames the meaning of reflective learning in the veterinary profession. Commonly utilised models of reflection in veterinary education include Gibbs reflective cycle, which is relevant for transitioning learners beginning university.^37^ Kolb's ELT^6^ has been used successfully for vets to promote reflection, assist clinical decision making and practical application of theory.^21^ However, there is some confounding work over models, while one set of evidence reported Kolb's ELT^6^ to be useful,^21^ others found models less useful.^3^, ^25^ This provides the scope to explore and develop these models for practical veterinary use.

It is notable that models to promote reflection are mainly used by individuals. As there is evidence that tutor support improves the level of reflection,^22^ facilitated or group reflection could provide the social aspect to learning, which can be missing when reflecting solo.^38^ One study discussed this, considering the key role colleagues have in supporting reflection.^28^ Being able to convert worry into useful reflection depended on the support, attitude and personal attributes of others on the team whose some graduates struggled to find.^28^ Team or group reflection could be helpful for development and has been linked to improved performance in medical students.^39^ It would be useful to investigate group or spoken reflection due to resistance to written reflection.^24^ Students seem to prefer spoken reflection, which could be a way of reducing assessment burden for students and staff compared to written essays, especially if these are peer assessed.

Schön's work on the practical application of problem solving for professionals and support for learners facing everyday problems is also applicable.^4^ Students initially carry out reflection‐on‐action (thinking and reflecting later), progressing to reflection‐in‐action (thinking and acting simultaneously) as they advance towards expertise.^4^ In Schön's observations, he analysed expert coaching novices who promoted deeper thinking by questioning their mentees.^4^ This compelled the student to stop and think of ways to solve previously unconsidered complications.^4^ In view of this, conversational reflection perhaps needs more exploration in our area. Introducing a social aspect to reflection, such as spoken peer group learning, could be a way of introducing new ideas and viewpoints, as in critical incident debriefs.^17^, ^40^

Postgraduate vets need time and supportive opportunities to formalise their reflections,^28^ which in the UK is provided via the Veterinary Graduate Development Programme.^41^ This requires new graduates to be mentored by experienced staff with time set aside for regular progression meetings. These meetings provide a social aspect to learning,^38^ which should provide a safe space to discuss and reflect on development. In the UK, newly graduated vets must complete regular reflection; it has been identified that a supportive workplace is essential for facilitating the development of reflection and diagnostic skills.^28^ Strengthening the importance of acceptance of reflection from experienced vets and a requirement to model this behaviour to provide a suitable environment for graduates.

Reflection does seem to be a worthwhile intellectual process for self‐improvement. In education, reflection is seen as a blend of different developing levels with apparently more acceptance of the process than the result.^42^ In contrast, for vets, reflection seems hierarchical, with critical reflection being awarded highest marks, as it is associated with personal development. This is understandable and perhaps this is driven by requirement to assess this learning, but it is questionable whether the process of assessment detracts from the authenticity or development of reflection. The question of what level of reflection attained in this hierarchy heeds once again to Schön's reflective practitioner and whether it should be sufficient for students to be on a self‐improvement journey, given that it is normal to develop at different rates.^4^ It would be interesting to discover whether these levels are important for success in first opinion practice.

Two publications distinguished the characteristics of these levels or types of reflection, which is useful.^23^, ^25^ However, types of reflection can be identified by individuals differently.^22^ Furthermore, confirmation of the features of reflection and in which setting each has most utility would be useful. Time is a common barrier to reflection.^42^ In a busy general practice time pressure might dictate rapid skills reflection, in contrast to the full academic appraisal required for critical reflection. This type of reflection may be no less useful in this setting and warrants further investigation for benefit and practicality.

Occasionally, reflection is mentioned in other work on non‐technical attributes, but it is uncommon to see detail in this topic. In an earlier review seeking evidence for veterinary professional non‐clinical competencies,^43^ the authors found that communication skills and professionalism were the only competencies that were explicit in all frameworks.^43^ Psychological constructs such as emotional intelligence, self‐awareness and self‐efficacy are sparsely mentioned.^43^ It is common to see reflection on communication skills.^3^, ^16^, ^21^ However, reflection permeates all professionalism and learning. For example, while acquiring attributes required by the developing veterinarian, such as ethics, values and evolving attributes of a professional.^33^ Currently, as work on reflection is increasing, it is becoming slightly more varied. As this learning is mandatory and ubiquitous in the UK, continued exploration of the utility of reflection and the effect it has at each stage of veterinary career would be beneficial.

A modest amount of empirical research is perhaps not surprising given that the approach is derived from philosophers such as Aristotle^44^ and Dewey.^29^ Nevertheless, for a concept widely used in veterinary curricula, there is a need for evidence of efficacy. It is acknowledged that a post‐positivistic perspective taken in this scoping review is at odds with the theoretical and interpretive nature of reflection as a concept. It is a theory or a model that does not exist outside the researcher, which logically therefore has more theoretical or opinion‐based evidence. One could argue this concept is in a different paradigm than this research methodology. It may seem contradictory to apply scoping review methods that could be considered a drawback of this approach. Nevertheless, we request that the reader flits across paradigms^45^; if we aim to educate from evidence, we consider this review to be justified.

The grey literature may be informative, as this is an emerging area, although it is beyond the scope of this resource. There may be many more examples of veterinary reflective practice that are unpublished and therefore are out of the scope of this review.

In summary, this review on reflection highlights the work in our field and emphasises gaps in research. It recognises that reflection is given weight in teaching and assessment at undergraduate and postgraduate levels, which necessitates the need for examination of this area in the veterinary sphere. It is acknowledged that in veterinary education, this is an emerging, academic topic. Furthermore, continued empirical work is required to determine how it can be best implemented and applied. However, it is recognised that due to the theoretical nature of reflection, this area is difficult to operationalise and therefore apply to education and clinical practice.

## AUTHOR CONTRIBUTIONS

Andrea Jones and Kate Cobb conceived and designed the project. Andrea Jones acquired the data. Andrea Jones, Kate Cobb and Gary England analysed and interpreted the data. Andrea Jones, Kate Cobb and Gary England wrote the paper.

## CONFLICTS OF INTEREST STATEMENT

The authors declare they have no conflicts of interest.

## FUNDING INFORMATION

The authors received no specific funding for this work.

## ETHICS STATEMENT

The authors confirm that the ethical policies of the journal, as noted on the journal's author guidelines page, have been adhered to. No ethical approval was formally required because this was a review article with no original research data. The study was granted approval by the Committee for Animal Research and Ethics at the School of Veterinary Medicine and Science, University of Nottingham (approval number: 3413 210727).

## Supporting information

Supporting Information

## Data Availability

The data that support the findings of this study are openly available.
