# Supplementary material for: A scoping review on the use of reflection and reflective portfolio learning in veterinary education
Source: Vet Rec Open. 2024 May 7;11(1):e279. doi: 10.1002/vro2.79 (PMC11075638; doi:10.1002/vro2.79)
Supplement: Supplementary file 1 — Supporting Information [file VRO2-11-e279-s001.pdf]

Supporting Information

Table S1 Table of 16 studies included in the review (in chronological order)

Reference number from main text	Reference	Method of evaluation	Participants	Aims	Outcomes
3	Adams CL, Nestel D, Wolf P. Reflection: A Critical Proficiency Essential to the Effective Development of a High Competence in Communication. J Vet Med Educ. 2006;33:58-64. DOI: 10.3138/jvme.33.1.58.	Mixed methods: Compared two case studies: content analysis of medical and veterinary student reflective assignments and assigned a frequency score for the levels of reflection attained by students	Medical and veterinary undergraduate students	To assess the level of reflection during application of this learning for a communication assignment	Medical students were assessed as being at a developmental level of reflection. Vets were self-aware and more capable of critical reflection but only saw the value of reflection for developing communication skills.
19	Boerboom TBB, Jaarsma D, Dolmans DHJM, Scherpbier AJJA, Mastenbroek NJJM, Van Beukelen P. Peer group reflection helps clinical teachers to critically reflect on their teaching. Med Teach. 2011;33:e615-23. DOI:10.3109/0142159X.2011.610840.	Mixed methods: Analysed level of reflection in written reflection reports and self-assessment questionnaire which evaluates teachers' performance	Veterinary educators	To explore effect of peer group reflection on reflective ability in response to student feedback using two feedback methods one with a peer reflection meeting and one without.	Student feedback was a good prompt for educators to plan strategies for improvement. Peer group reflection enabled a higher level of reflection
17	de Groot E, Endedijk M, Jaarsma D, van Beukelen P, Simons RJ. Development of critically reflective dialogues in communities of health professionals. Adv Health Sci Educ Theory Pract. 2013;18:627-643. DOI: 10.1007/s10459-012-9403-y.	Mixed methods: analysed audio recordings of practicing vets' critically reflective work dialogues - numerically categorised and interpreted perspectives of participants	Veterinary professionals	To explore whether aspects of critically reflective dialogue change over time	Some communities became more open about mistakes over time, a finding related to increased trust. Others perceived change in their development but quantitatively stayed the same. High level reflection did occur, but it was rare
20	de Groot E, Endedijk MD, Jaarsma ADC, Simons PRJ, van Beukelen P. Critically reflective dialogues in	Mixed methods: content analysis of audio recordings of	Practicing veterinary professionals	To apply a developed framework to study	According to the authors classification most modes of communication were non-

	learning communities of professionals. Studies Cont Educ. 2014;36:15-37. DOI: 10.1080/0158037X.2013.779240.	reflective group meetings, resulting in a framework to explore critically reflective dialogues.		interactions in critical reflective work dialogues	interactive, individual and non-reflective.<br>They conclude that interventions to improve the quality of interactions should focus on promoting reasoning and reflection between members.
21	Meehan MP, Menniti MF. Final-year veterinary students' perceptions of their communication competencies and a communication skills training program delivered in a primary care setting and based on Kolb's Experiential Learning Theory. J Vet Med Educ. 2014;41:371-383. DOI: 10.3138/jvme.1213-162R1	Mixed methods:<br>likert questionnaire and text analysis, pre and post a communication skills training programme	Veterinary undergraduate students	To investigate students' perceptions of a communication skills training programme implemented within a primary care setting	Concluded Kolb's E.L.T. helps with reflection on communication skills within primary care practice
26	Khosa DK, Volet SE, Bolton JR. Making clinical case-based learning in veterinary medicine visible: analysis of collaborative concept-mapping processes and reflections. J Vet Med Educ. 2014;41:406-417. DOI: 10.3138/jvme.0314-035R1	Mixed methods:<br>student assignment scores and video coding (for engagement and knowledge construction at a high or low level)	Veterinary undergraduate students	To explore students' accounts and reflections of collaborative concept mapping to enhance understanding of a clinical case.	Small mention of reflection in this paper but provides evidence to support concept mapping as a method to order thoughts and link concepts
22	Silva-Fletcher A, May H, Magnier KM, May SA. Teacher development: A patchwork-text approach to enhancing critical reflection in veterinary and para-veterinary educators. J Vet Med Educ. 2014;41:146–54. DOI: 10.3138/jvme.0813-110R	Qualitative: analysed reflective essays	Veterinary educators	To assess utility of the patchwork-text approach for the development of reflection in veterinary educators with a science background who had not used a reflective approach before. Also, to investigate whether reflection could be graded using these essays	The patchwork text (short essays) method was found to be useful to develop reflective ability. Reviewing the essays with tutor feedback increased reflective ability<br>It was possible to grade but it was not straightforward to ascertain levels of reflection
13	May SA, Kinnison T. Continuing professional development: learning that leads to change in individual and collective clinical practice. Vet Rec. 2015;177:13. DOI: 10.1136/vr.103109	Qualitative: analysis of essays for Cert AVP professional key skills module	Postgraduate veterinary students	To explore the effect of an outcomes-based approach to continued professional development via participants' reflective essays	Found proposed behavioural changes as a result of the professional and business skills module which positively impacted wider practice stakeholders (team, owners, patients) as well as participants

14	Kinnison T, May S. Continuing professional development: researching non-technical competencies can support cognitive reappraisal and reduced stress in clinicians. Vet Rec. 2017;181:266. DOI: 10.1136/vr.104426.	Qualitative: analysis of essays for Cert AVP professional key skills module	Postgraduate veterinary students	To explore the change in emotion (negative to positive) as a result of reflective summaries produced during a Professional Key Skills module	After reflection participants understood the importance of professional topics to job satisfaction. The module stimulated stress coping responses and more reasonable expectations of themselves, more work appreciation & reduced stress. The focus on reflection changed emotion from anxiety to feeling more content. Resulting behaviour changes benefited multiple stakeholders including the students and their wider practice team.
15	Dixon WHR, Kinnison T, May SA. Understanding the primary care paradigm: an experiential learning focus of the early veterinary graduate. Vet Rec. 2017;181:480. DOI: 10.1136/vr.104268.	Qualitative: Analysis of essays for Cert AVP professional key skills module	Postgraduate veterinary students	To find which challenges newly graduated vets face by looking at reflective essays on a puzzling case. To ascertain whether challenges were related to technical or non-technical aspects of the case.	The main challenges facing newly graduated vets were difficulty with clinical decision making, continuity of care, time pressures and support in the transition to practice. Lack of experience in commonly encountered cases in general practice was reported leading authors to suggest greater focus on primary care is important in veterinary education.<br>Reflection helped identify challenges which is helpful for professional development
28	Warman SM. Experiences of recent graduates: reframing reflection as purposeful, social activity. Vet Rec. 2020;186:347. DOI: 10.1136/vr.105573	Qualitative: semi-structured interviews of newly graduated vets studying the RCVS PDP	Recently graduated vets	To explore how recently qualified veterinary surgeons engage in reflection during their PDP	The importance of social reflection and finding a way to formalise day to day reflection were found to be important. A supportive environment is essential for reflective practice, concerns around judgement arose when reflective practice is evaluated as part of post graduate training.
23	Armitage-Chan E, Reissner S. How do veterinary students engage when reflecting on their development	Qualitative: Thematically analysed student essays to	Undergraduate veterinary students	Qualitative exploration of student essays to	Defines levels of reflection for veterinary area which are described

	towards being veterinary surgeons? Vet Rec. 2020;187:e77. DOI: 10.1136/vr.105692	identify critical and core reflection.		synthesise theoretical framework for reflection	in terms of resources, practices and outcomes.
16	Proudfoot KL, Ventura BA. Impact of a Frame Reflection Assignment on Veterinary Student Perspectives toward Animal Welfare and Differing Viewpoints. J Vet Med Educ. 2021;48:361-372. DOI: 10.3138/jvme.2019-0123	Mixed methods: thematic analysis of student responses to the survey questions and quantitative to determine relationships between themes and sex or career area	Undergraduate veterinary students	Evaluation of frame reflection to develop communication skills through increasing students' understanding of different perspectives and values regarding controversial animal welfare topics.	Frame reflection was useful to facilitate communication and to see other viewpoints on animal welfare Female students demonstrated more judgement, a lack of shared values, and a desire to change others' point of view
24	Duret D, Terron-Canedo N, Hannigan M, Senior A, Ormandy E. Identifying the Barriers to Incorporating Reflective Practice into a Veterinary Curriculum. J Vet Med Educ. 2021;49:454-461. DOI: 10.3138/jvme-2020-0040	Qualitative: thematic analysis of focus groups	Undergraduate veterinary students	Investigate student understanding of reflection and perceptions of the portfolio assessment	Found students did not perceive the value of reflection and were resistant to personal reflections being judged
25	Armitage-Chan E, Reissner S, Jackson E, Kedrowicz A, Schoenfeld-Tacher R. How Do Veterinary Students Engage When Using Creative Methods to Critically Reflect on Experience? A Qualitative Analysis of Assessed Reflective Work. J Vet Med Educ. 2021;49:632-640. DOI: 10.3138/jvme-2021-0070.	Qualitative: analysed student essays using a narrative approach	Undergraduate veterinary students	To explore how creative methods develop critical reflection	Found creative methods can be used to facilitate critical reflection in some cases. Provides further definition of reflection in the veterinary area.
27	Warman SM. The individual in the system: The role of affect in recent veterinary graduates' reflective activity. Vet Rec. 2021;e304. DOI: 10.1002/vetr.304	Qualitative: thematic analysis of semi-structured interviews	Recently graduated vets	To explore the influence of feelings, emotion and mood on recent graduates' experience of reflection	Feelings, emotions and workplace mood influence the engagement with and outcomes of reflective activity. Support from others in the practice was helpful whereas discontent in the workplace had the opposite effect.
